# Consistency of thresholds for eutrophication assessments, examples and recommendations

**DOI:** 10.1007/s10661-021-09189-6

**Published:** 2021-09-29

**Authors:** D. Topcu, U. Brockmann

**Affiliations:** 1 Dept. Biogeochemistry, Institute for Geology, Hamburg University, Hamburg, Germany; 2 Institute for Meteorology, Hamburg University, Hamburg, Germany

**Keywords:** Assessments, Thresholds, Eutrophication, North Sea, Baltic Sea

## Abstract

International harmonisation of management goals for eutrophication processes in coastal waters, requiring reduction of discharges and depositions of nutrients and organic matter, needs coordinated assessments and measures. This is especially necessary in open areas, connected by currents and mixing processes with trans-boundary exchanges. Management goals, defined nationally as local thresholds for nutrients and chlorophyll-a, had been applied recently (2006–2014) within international eutrophication assessments in the North Sea (OSPAR) and Baltic Sea (HELCOM). Consistency of thresholds for nitrogen nutrients and chlorophyll-a concentrations is tested by mixing diagrams and correlations between nitrogen nutrients (total and inorganic nitrogen) and chlorophyll-a. Results indicate mean consistent relations, but single deviations as in the continental coastal water of the North Sea surpassed means by a factor up to 5 for chlorophyll-a in relation to inorganic nitrogen. Thresholds differed across national borders significantly. Correlations of thresholds and assed data reflect the degree of regional deviations by comparison. Consistency of regionally applied thresholds can be achieved stepwise, by application of regionally correlated means, by adaptation to mixing and parameter relations, and finally by relations of thresholds to natural background concentrations. By this, consistency of international assessments can be improved generally, allowing coordinated management of open coastal waters.

## Introduction

Eutrophication is still one of the most harmful threats for coastal waters (Cloern, 2001). Population growth and further coastal urbanization will probably exacerbate eutrophication and coastal hypoxia (Doney, 2010). “Eutrophication is a significant issue in all European regional seas” maintained by a significant gap between the current eutrophication status and the reduction in inputs achieved so far in some areas (EC, 2019). The coastal ecosystems in the North Sea and Baltic Sea are still affected significantly, despite considerable regional reductions of nutrient concentrations in main rivers discharging to the North Sea (Nienhuis, 2002a; Nienhuis, 2002b a, b, Duarte, 2009, Brockmann et al., 2018, Greenwood, 2019) and to the Baltic Sea (HELCOM, 2018a, 2018b, 2018c). Atmospheric nitrogen depositions had been reduced as well (Prospero, 1996; Rendell et al., 1993; Ruoho-Airolaet al., 2012), but ongoing river discharges are still elevated, and eutrophication effects are intensified by the climate change (Andersson et al., 2015; Duarte, 2009; Rabalais et al., 2009).

Multi-national conventions for protection of marine environments in northern Europe are based on coordinated monitoring, assessments, and management by the member states (OSPAR, 2017, HELCOM, 2018a, 2018b, 2018c, 2018a, EC, 2005). OSPAR (Oslo-Paris Commission) is responsible for the NE Atlantic, HELCOM (Helsinki Commission) for the Baltic Sea, the EC-WFD (European Water Frame Work Directive) for European coastal waters, and the EC-MSFD (European Marine Strategy Framework Directive, EC, 2008) for European offshore waters. Accordingly, the continuing eutrophication problems were reflected by recent international assessments (HELCOM, 2018a, 2018b, 2018c; OSPAR, 2017). In the North Sea, the largest “problem area” was identified along the continental coast from Belgium to Danish and Swedish waters during recent assessments between 2006 and 2014. The main drivers were nutrient discharges by rivers. Atmospheric nitrogen inputs to the North Sea were reduced by 30% since 1990 (OSPAR, 2017). The spatial extent of problem areas decreased since 2003 by 40%. Decreasing nutrient discharges to the Baltic Sea were reported for 2011–2016 (HELCOM, 2018a, 2018b, 2018c) but recent stagnations keeping eutrophication at elevated levels. Ninety-seven percent of the open Baltic Sea were still assessed to be below good eutrophication status and 86% of the coastal waters. Levels of nutrient indicators were indicated generally as “furthest away from good status”.

Internationally agreed assessment procedures were basically performed nationally, mostly differentiated for inshore, coastal and offshore areas, based on regular seasonal/annual monitoring, including vertical profiles of salinity, temperature, inorganic nutrient concentrations at least at the surface, partly total nutrients, chlorophyll-a (all parameters periodically inter-calibrated), often phytoplankton composition, Secchi depth, oxygen concentrations, and zoobenthos at the bottom (Karydis, 2009). Surface concentrations of nutrients and chlorophyll-a were assessed in the North Sea and Baltic Sea seasonally. Considering that inorganic nutrient concentrations are the highest at the surface during winter, assessment period was restricted to the winter (XII-II) (HELCOM, 2013, 2018a, 2018b, 2018c; OSPAR, 2017). Biological components, like chlorophyll-a, reached the highest concentrations during the assessed growing season, mostly III-X in the North Sea and IV-X in the western and for shorter periods in the north-eastern Baltic Sea (Feistel & Nausch, 2008; HELCOM, 2018a, 2018b, 2018c; OSPAR, 2017). Total nitrogen was assessed mainly during all seasons, due to its smaller and variable seasonal differences. Phytoplankton is dependent from available nutrient concentrations, indicated by correlations between chlorophyll-a concentrations and nutrients, especially with total nitrogen (Nielsen et al., 2002; Smith, 2006; Tett et al., 2003). Chlorophyll-a is a widely used indicator for phytoplankton abundance and biomass in coastal and estuarine waters because of direct relations to phytoplankton biomass and thus is widely used as an indicator of water quality, where high concentrations show eutrophication and low concentrations indicate nutrient limitation, especially by nitrogen (Howarth & Marino, 2006; Smith, 2006). Since chlorophyll-a concentrations affect Secchi depths (Fleming-Lehtinen & Laamanen, 2012) or macro-zoobenthos biomass (Beukema et al., 2002; Brockmann et. al, 2018; Hargrave & Peer, 1973), this parameter has a central function within ecological assessments. By assessing local inter-annual means, combining a couple of years, and integrating local and inter-annual variations, effects by variable precipitation, changing land uses, and flow conditions to coastal waters were reduced. However, the variability within ecosystem processes was considered by assessing of regional maxima additionally, indicating e.g. specifically phytoplankton blooms or maximum oxygen depletion. Analyses of trends were included in regional assessments by assimilation of previous assessment periods.

For eutrophication assessment procedures, threshold values of individual nutrients and other ecosystem indicators have been developed to help manage pollution loading into coastal waters and to better understand the effects of elevated nutrients in coastal ecosystems. Thresholds applied by OSPAR (2017) defining the border between”non-problem” and “problem” area correspond to HELCOM (2018a, 2018b, 2018c) thresholds good/not good, to WFD thresholds “good/moderate” (EC, 2009a, 2009b), or to MSFD definitions where “non-problem” is like the good environmental status (Andersen et al., 2004; EC, 2009a, 2009b). Accordingly, thresholds were transferred between the assessment systems (HELCOM, 2018a, 2018b, 2018c). However, a Pan-European assessment of eutrophication status has not been attempted, partly because of a lack of science-based threshold values (EC, 2019). Correspondingly, assessment levels, thresholds, or reference values had been modified several times by OSPAR member states since the first common assessment in 2003, recently according to the need for coherent assessments with the WFD and MSFD, based on new knowledge and/or historical data. OSPAR started checks of threshold consistency considering mixing conditions and correlations between nutrients and chlorophyll-a (OSPAR, 2017). HELCOM thresholds for coastal areas have been inter-calibrated under the WFD for some indicators or have been set through national decisions, based on various methods: reference sites, historical data, modelling, and expert judgement. Some thresholds are still being tested (HELCOM, 2006, 2018a, 2018b, 2018c). Different to the other assessment methods, by HELCOM, eutrophication ratios (assessment value/threshold) were applied to arrive at a common scaling for all indicators. Local reference values applied within OSPAR or HELCOM were mostly identical with parallel assessments for WFD and MSFD in the same area.

Especially in open coastal areas, connected by currents and tidal mixing processes with frequent exchanges of water masses between neighbouring areas assessed nationally within multi-national conventions in the North Sea and Baltic Sea (EC, 2005; HELCOM, 2017; OSPAR, 2017), coordinated measures are needed, based on assessments with consistent thresholds (Almroth & Skogen, 2010). Related to European assessments of eutrophication loads, the applied thresholds showed high diversity (Dworak et al., 2016), requiring consistent improvements, based on natural processes like mixing and ecosystem interactions, as proposed by this paper. Since mean local thresholds were defined independently for the nationally assessed coastal and offshore areas, inconsistent results across national borders could not be avoided, and significant differences were observed, contradicting to recent continuous gradients of salinity, nutrients, and chlorophyll-a (HELCOM, 2018a, 2018b, 2018c; OSPAR, 2017). For these reasons, applied thresholds were not comparable across the regions as well. By relation of nutrients or chlorophyll-a thresholds to salinities, effects of variable hydrodynamic processes like mixing can be compensated (Almroth & Skogen, 2010; Greenwood et al., 2019; Topcu et al., 2009). Additional inconsistencies between applied thresholds occurred in quantitative relations between interacting ecosystem components, such as between nitrogen nutrients and phytoplankton (chlorophyll-a) as examples. Despite the efforts to define thresholds at national (OSPAR, 2017) or regional levels (HELCOM, 2007), applied thresholds have been reported without definition of their specific deduction methods. The relative deviations of applied thresholds were investigated by mixing diagrams for selected parameters as for nitrogen nutrients and by correlations between interacting parameters as nitrogen nutrients and chlorophyll-a, in the North Sea for locally applied values (OSPAR, 2017) and in the Baltic Sea as basin means (HELCOM, 2007). For the North Sea, mixing diagrams of dissolved inorganic nitrogen (DIN) and for the Baltic Sea of total nitrogen (TN) has been chosen, because nitrogen was often limiting coastal phytoplankton growth (Howarth & Marino, 2006). Both parameters were regionally significantly correlated with assessed parameters like chlorophyll-a or Secchi depth (Fleming-Lehtinen & Laamanen, 2012; Nielsen et al., 2002; Smith, 2006; Tett et al., 2003).

Based on the assessment results (OSPAR, 2017 and HELCOM, 2018a, 2018b, 2018c), further harmonisations of thresholds are proposed. Since data sets of eutrophication parameters are most robust due to regular monitoring and inter-calibrations for combined assessments, they could serve as base for holistic classifications of coastal waters (HELCOM, 2018b). Since threshold values are still discussed by OSPAR (2017) and HELCOM (2018a, 2018b, 2018c), improvements of coordinated assessments by consistent assessment levels could well be considered. This would support coordinated reductions measures of nutrient discharges by more effective sewage plants (Sartorius et al, 2011), changes of agriculture by applying less fertiliser (Andersen, 2017; Desmit, 2018; Pavlidou, 2015), and accompanied by continuing reduction of atmospheric nitrogen depositions (Bartnicki et al., 2017).

## Methods

Analyses and recommendations were focussed on main areas in the North Sea and Baltic Sea, not considering the huge variability (Herrero et al., 2019) of the numerous small-scaled WFD-areas, e.g. along the Baltic Sea coasts > 240 areas (HELCOM b, 2018a, 2018b, 2018c). Thresholds for nitrogen nutrients and chlorophyll-a have been transferred as examples from recent assessments in the North Sea and Baltic Sea (HELCOM, 2017; OSPAR, 2017), together with the assessed recent surface concentrations (2006–2014) and combined with salinities from the ICES database which was the main data source for the assessments as well. For the North Sea, data were extracted from the integrated report (OSPAR, 2017), supplemented by data from national reports for local values. Because of nearly constant DIN concentrations of basin means in the Baltic Sea, TN was chosen here, reflecting gradients in mixing diagrams, but TN had not been assessed in some westerly offshore areas (areas 4–7, see Fig. 3).

Thresholds, defined in the North Sea mainly for local inshore waters, coastal and national offshore waters, and accumulated as basin means in the Baltic Sea, were combined with local salinities from national reports or ICES data for mixing diagrams. Even in the Baltic Sea with its weak salinity gradients (Feistel et. al., 2008), historical mixing diagrams can be applied due to long-lasting nutrient changes since pristine conditions with low nutrient discharges (Gustafsson et al., 2012; Savchuk et al., 2008). Thresholds and recent data were transformed for correlations to square means of 145.23 km^2^ as grids, approaching ICES boxes in North Sea, or were compiled as basin means in the Baltic Sea from a HELCOM compilation (HELCOM, 2017).

Inshore and coastal thresholds for Norway and Denmark and inshore values from the UK were not included in the recent assessment because of missing data points and have been transferred from the foregoing assessment (COMP 2, OSPAR, 2008). In addition, only DIN threshold values in coastal waters were included from France. Thresholds for the coastal Baltic Sea areas had been inter-calibrated according to the Water Framework directive for chlorophyll before or have been set through national decisions as for nutrient concentrations (HELCOM, 2017). The same holds for North Sea thresholds (OSPAR, 2017).

**Fig. 1:**
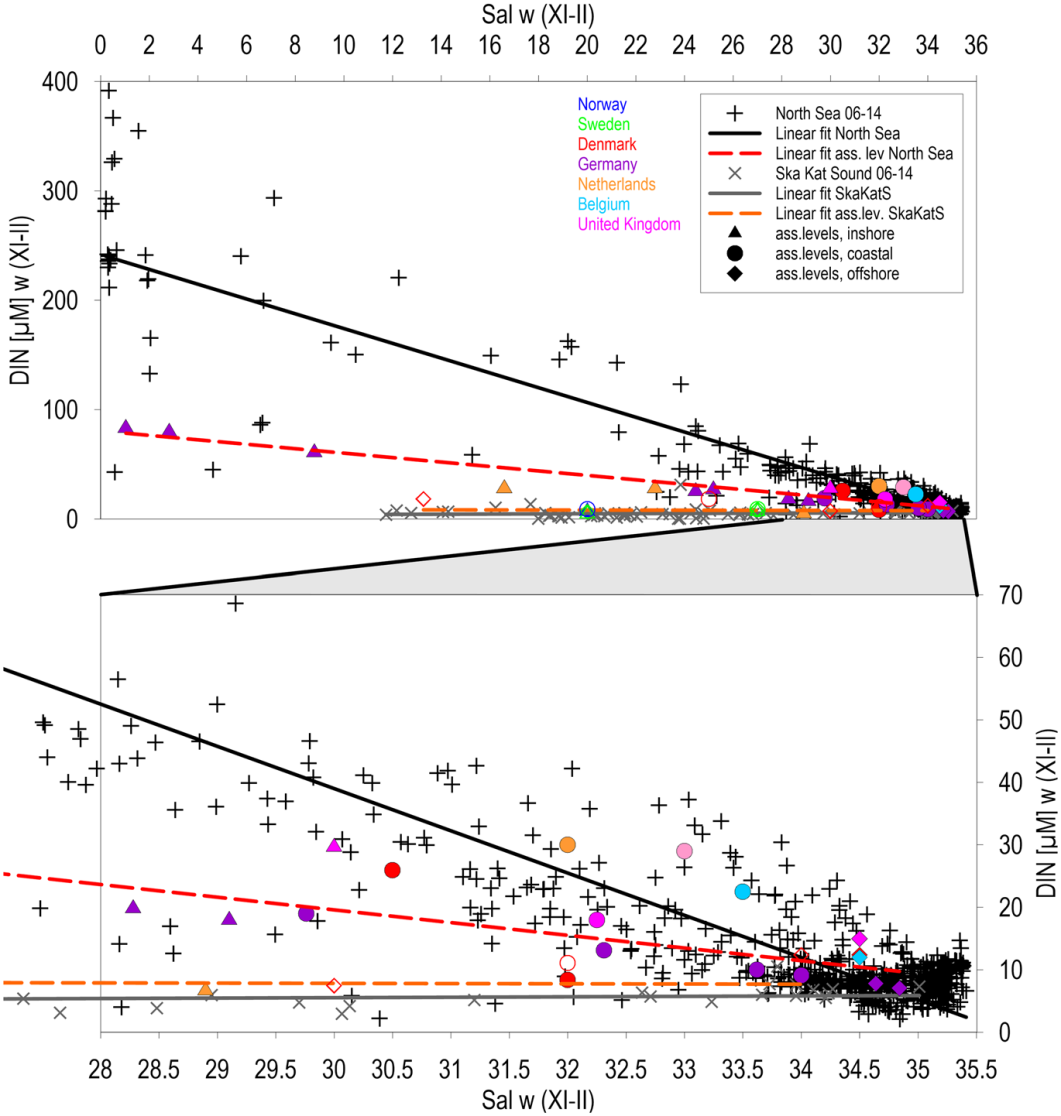
Mixing diagrams of DIN square means (145.23 km^2^) in the North Sea and the combined Skagerrak, Kattegat, and Sound (2006–2014), supplemented by mixing diagrams of thresholds (main North Sea:Y =  −6.757 × X + 241.72, n = 695, R^2^ = 0.86, α < 0.1% and Y =  −2.032 × X + 80.56, n = 28, R^2^ = 0.89, α < 0.1%, Skagerrak/Kattegat: Y = 0.069 × X + 3.469, n = 66, R^2^ = 0.011, α > 5%, Y =  −0.035 × X + 8.88, n = 18, R^2^ = 0.009, α > 5%), open symbols for thresholds. Netherlands offshore areas with 15 µM DIN are covered by an identical UK value

Offshore thresholds for TN (9.65 µM) and DIN (7.94 µM) were calculated as regional means from recent data at salinities 34.5–35 (PSU) for marine mixing end-members of the North Sea, assuming long-lasting mixing processes and reflecting natural background concentrations (Fig. 1), and have been applied in mixing diagrams. Maps of thresholds have been plotted with the reported data for national areas in the North Sea or basins in the Baltic Sea (HELCOM, 2017; OSPAR, 2017) and additionally transferred to recent salinity gradients, applying presented correlations between thresholds and salinities. For offshore North Sea areas (salinity > 34.5), recent data have been taken as final possible thresholds. Square means for mapping included 3275 km^2^ each. For the main North Sea, the Skagerrak/Kattegat/Sound area was calculated separately due to diverging mixing diagrams of thresholds and recent data. Some western areas in the Baltic Sea had not been assessed for TN. Means, mixing diagrams, maps, and parameter correlations has been calculated and plotted for applied thresholds (HELCOM, 2017; OSPAR, 2017) and recent data by the software “Surfer” (Golden Software). By this, consistency of thresholds was tested in relation to mixing processes by comparison of single values with means and recent data. Consistency in relation to interacting ecosystem processes was tested by selected parameter correlations, such as between DIN and chlorophyll-a in the North Sea and between TN and chlorophyll-a in the Baltic Sea.

Thresholds and recent data were taken from identical seasons, for the North Sea DIN during winter (XI-II), chlorophyll-a during growing seasons (III-X), for the offshore Baltic Sea nutrients were taken as annual means and chlorophyll-a during growing season (VI-IX). However, despite maxima of DIN concentrations in the North Sea during winter and chlorophyll-a maxima during the growing season, both parameters were regionally correlated (Brockmann et al., 2018) because surface data were dominated by horizontal gradients. Additionally, seasonal differences were reduced by primary production during winter because phytoplankton does not rest completely during winter (Zingone et al., 2010), especially not in shallow areas (Brockmann & Wegner, 1995). For this reason, thresholds of nutrients and chlorophyll-a were related to these inter-seasonal parameter correlations as well. Data for correlations between TN and chlorophyll-a in the Baltic Sea were transferred from assessments of basin means directly (HELCOM, 2018a, 2018b, 2018c).

## Results

Mixing diagrams of dissolved inorganic nitrogen (DIN) was significant for recent surface data and the applied thresholds in the main North Sea including estuaries, increasing in freshwater to recent 242 µM DIN and thresholds to 81 µM (Fig. 1). Mean thresholds for DIN had been defined 67% below recent freshwater means, decreasing offshore to 5.9 µM DIN at a salinity of 34.5, corresponding to 6.1 µM DIN as recent offshore mean. In the main North Sea, most elevated thresholds were indicated in relation to means for inshore waters in Germany and UK, for coastal waters in the Netherlands, Belgium, and offshore for the Netherlands, Belgium, and UK. Referring to the most significant deviations, DIN thresholds of 30 µM, applied in the coastal waters of the Netherlands, surpassed mean thresholds by a factor 2, and the applied 22.5 µM in coastal waters of Belgium by a factor of 1.8 (Fig. 1). Mean thresholds topped recent mean offshore concentrations of 8.60 µM DIN with 10.5 µM DIN by 21.6% at a salinity of 34.5. Offshore thresholds of 15 µM DIN, applied by UK and the Netherlands, surpassed the combined mean by a factor of 1.4 or 34.5% and recent means (2006–2014) by 1.74 or 74.4%. Mixing diagrams were completely different in the Skagerrak–Kattegat–Sound region, connected with the succeeding Norwegian coast by the Baltic Sea outflow, from those in the main North Sea, due to low recent DIN concentrations (mainly 3.5 µM). The applied thresholds corresponded generally regional to these low values, but means remained in the Skagerrak/Kattegat nearly double of recent means (8.2 µM at a salinity of 20 and 4.8 µM recent). Correlations within the Skagerrak/Kattegat area were due to low and similar DIN concentrations not significant.

**Fig. 2:**
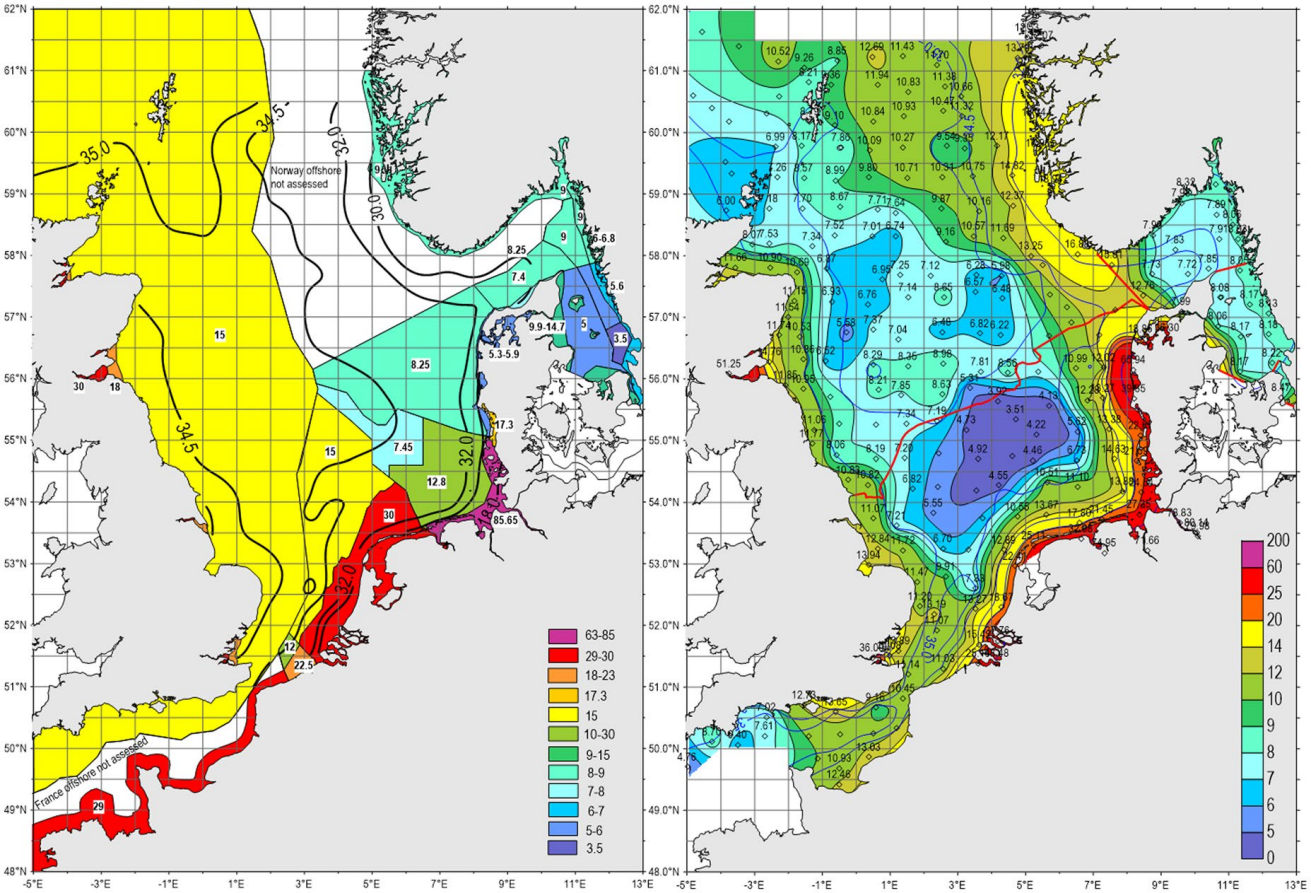
Thresholds of winter-DIN applied during COMP-3 in the North Sea (OSPAR, 2017) left, and mean thresholds calculated from mixing diagrams (Fig. 1), converted to square means of 3275 km^2^ and related to recent mean salinities right. For the Skagerrak/Kattegat, the regional specific mixing diagram was applied

Thresholds of DIN caused significant inconsistencies along national assessment boarders, especially between Germany and the Netherlands with 12.8 and 30 µM and offshore between Germany with 7.45 µM DIN and the Netherlands and UK applying both 15 µM (Fig. 1) (Fig. 2, left), but recalculated mean threshold gradients, transferred from mixing diagrams (Fig. 1), and related to recent salinity gradients, resulting in consistent distributions with highest values along the coasts (Fig. 2, right). Recent offshore DIN concentrations (at salinities > 34.5), applied as thresholds, remained below 10 µM and indicated the lowest values in the Dogger Bank region (< 5 µM). In comparison to the patchwork of applied thresholds between 7.4 and 30 µM DIN along the continental coast (at salinities < 32), the recalculated thresholds did not differ at national borders, following salinity gradients. Since applied German thresholds for DIN were inshore above the mean mixing line and values in the Netherlands below (Fig. 1), the recalculated threshold gradients partly increased or decreased accordingly in relation to the applied original values (Fig. 2). However, the differentiation between inshore and coastal waters was nationally deviating, and thresholds in inshore waters were partly characterised by steep gradients within the estuaries. The original extension of the 30-µM threshold area for coastal waters in the Netherlands and Belgium was by the relation to salinity gradients significantly reduced. Recalculated thresholds increased along German/Danish coastal waters at salinities < 32 to > 20 µM DIN (Fig. 2). The continuous belt of thresholds between 12 and 25 µM DIN reflected along the continental coast the transport of nutrients by the coastal current (Brockmann et al., 2018; Otto et al., 1990).

**Fig. 3:**
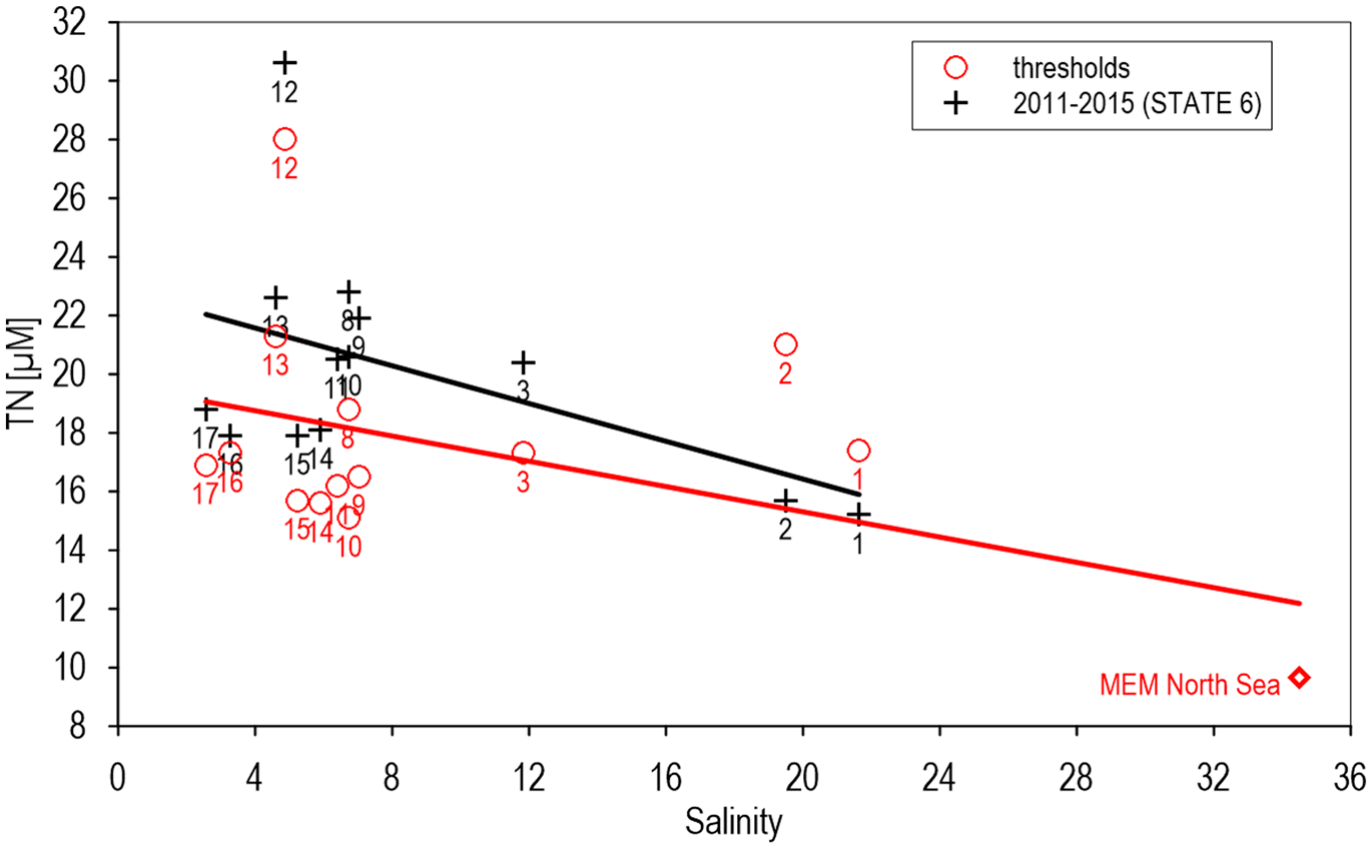
Correlations of basin means of TN (2011–2015) and thresholds from STATE 6 Indicator reports in the Baltic Sea (HELCOM, 2017). MEM marine end-member in the North Sea (9.65 µM). Kattegat (1), Great Belt (2), The Sound (3), Kiel Bay (4), Bay of Mecklenburg (5), Arkona Sea (6), Bornholm Sea (7), Gdansk Basin (8), Eastern Gotland Basin (9), Western Gotland Basin (10), Northern Baltic Proper (11), Gulf of Riga (12), Gulf of Finland (13), Åland Sea (14), Bothnian Sea (15), The Quark (16), Bothnian Bay (17). Areas 4–7 were not assessed for TN. (Y =  −0.189 × X + 19.53, n = 14, R^2^ = 0.177, α > 5%, Y =  −0.323 × X + 22.88, n = 13, R^2^ = 0.238, α > 5%)

There are only weak, insignificant mixing correlations in the Baltic Sea, due to small salinity gradients and reduced data by application of basin means. However, all mixing diagrams, those of recent (2011–2016) basin means and those of thresholds, reflected decreasing tendencies of TN concentrations at increasing salinities (Fig. 3), indicating river discharges as the main nutrient sources within the Baltic Sea, calculated from basin means: 23 µM TN recently and 19.5 µM for the combined thresholds at zero salinity.

**Fig. 4:**
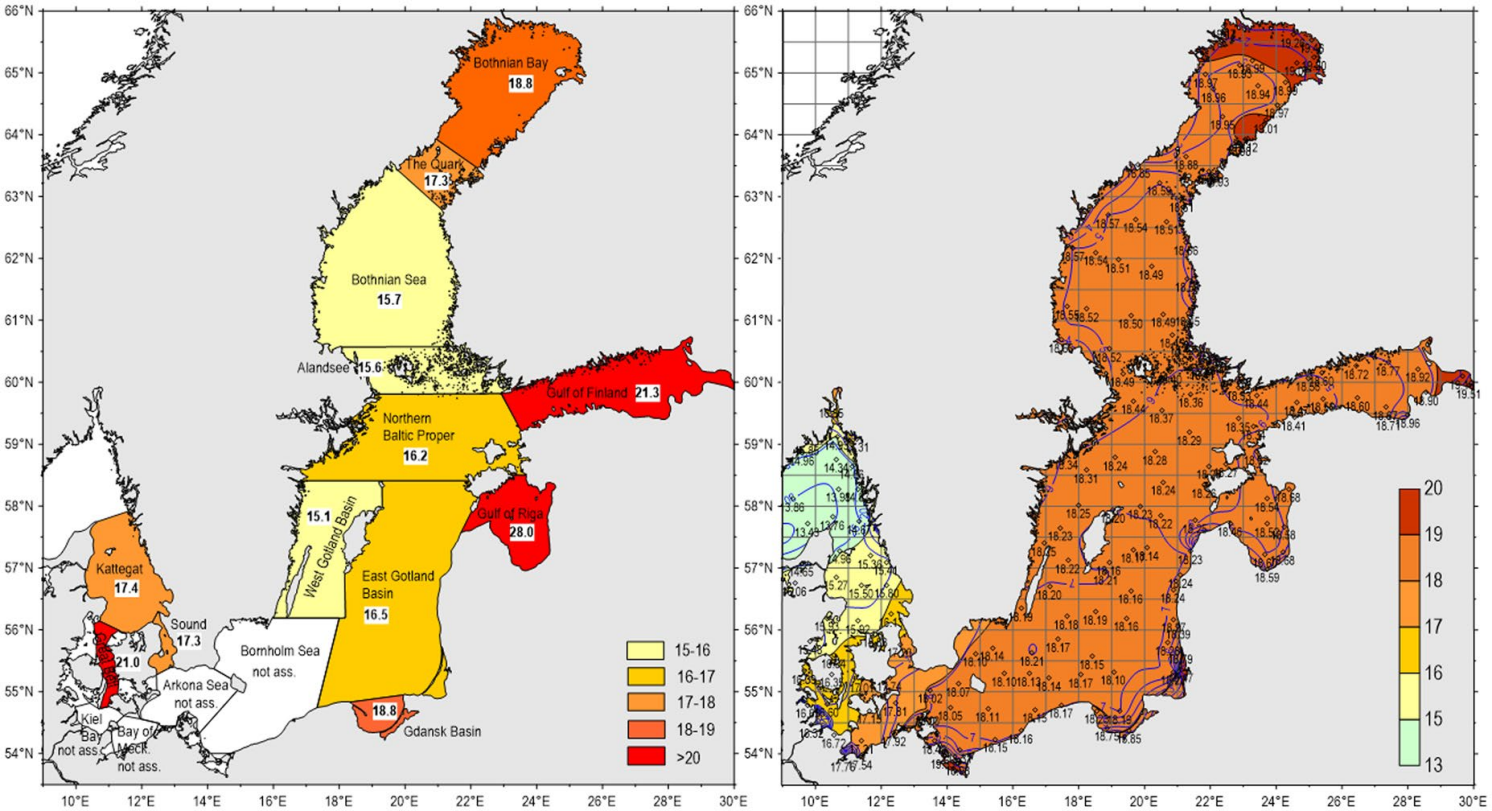
Applied (left) and salinity-adapted (TN µM =  −0.189 × salinity + 19.526, right) TN thresholds (µM) in the Baltic Sea. STATE-6 (HELCOM, 2017) (mean salinity 2006–2014)

Especially thresholds of TN in the Gulf of Riga (12), Gulf of Finland (13), and Great Belt (2) were elevated in relation to the mean mixing lines of thresholds and recent basin means (HELCOM, 2017). Thresholds for the Gulf of Finland (13) were placed in recent means. The most significant deviations from mean TN thresholds (Fig. 3) were compiled in Table 1. The responding regional distributions of basin mean thresholds (Fig. 4 left) revealed a patchwork between 15.1 and 28.0 µM TN. The strongest difference reached a factor of 1.7 between the Gulf of Riga with estimated 28 µM to 16.5 µM TN in the adjacent East Gotland Basin. Based on correlations between TN and salinity (Fig. 3), the thresholds for TN could be adapted to salinity gradients for the Baltic Sea as well, corresponding to mixing gradients between 15 and 19 µM TN, reflecting long-term mixing with the North Sea. In comparison to the original patchwork surpassed the salinity-related threshold means the regional means in the Bothnian Sea, Åland Sea, and western Baltic Proper.

**Table 1:** Most elevated thresholds for the selected parameters, national for North Sea (Figs. 1 and 5), and basin-related means (numbers) for the Baltic Sea (Figs. 3 and 6). TN was not assessed in the North Sea as a basic parameter

Parameter	TN	DIN	Chl./DIN	Chl./TN
Mixing diagrams	002, 012	NL, BE, UK		
Chlorophyll correlations			NL, BE, UK, 012, 009	009

002: Great Belt, 009: Eastern Gotland Basin, 012: Gulf of Riga

**Fig. 5:**
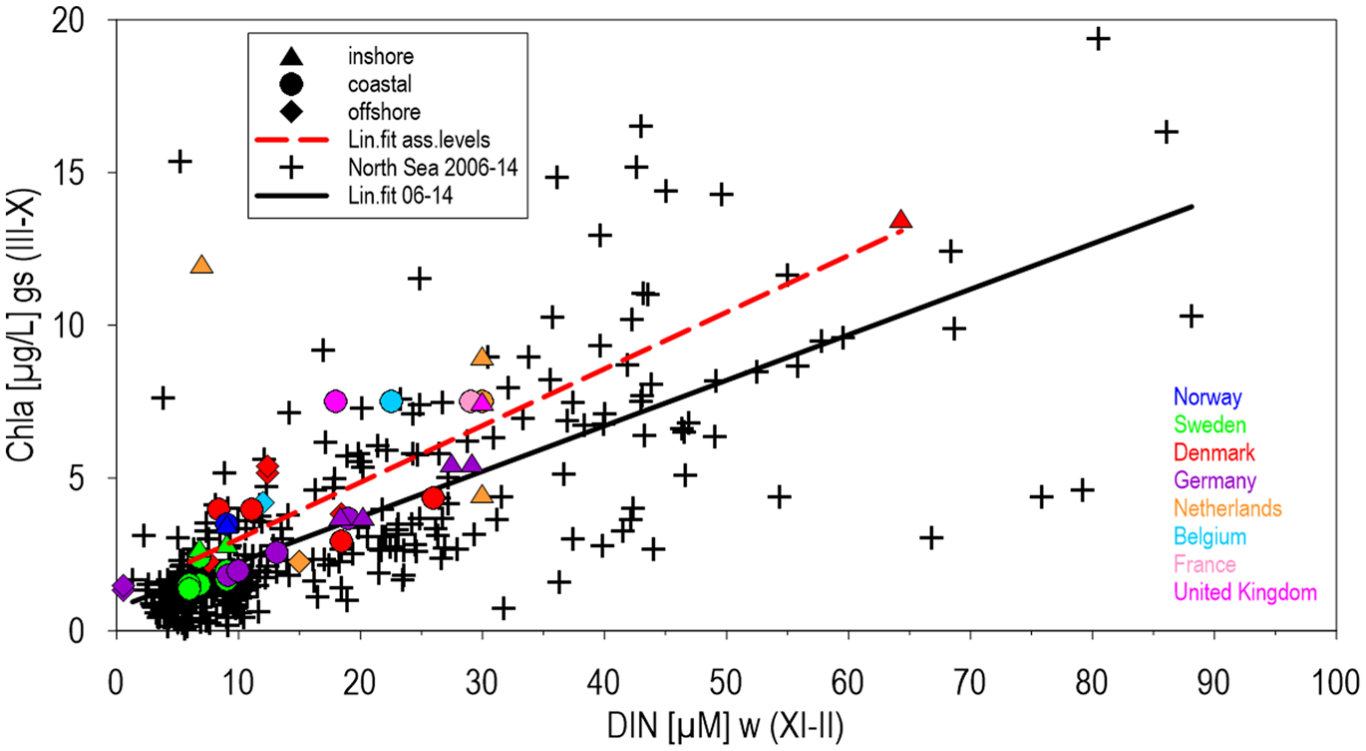
Correlations between square means (145.23 km^2^) of chlorophyll a during growing sesaons and DIN in the North Sea during winter 2006–2014. Y = 0.149 × X + 0.747, n = 281, R^2^ = 0.534, α < 0.1%, Y = 0.189 × X + 1.051, n = 44, R^2^ = 0.532, α < 0.1%

**Fig. 6:**
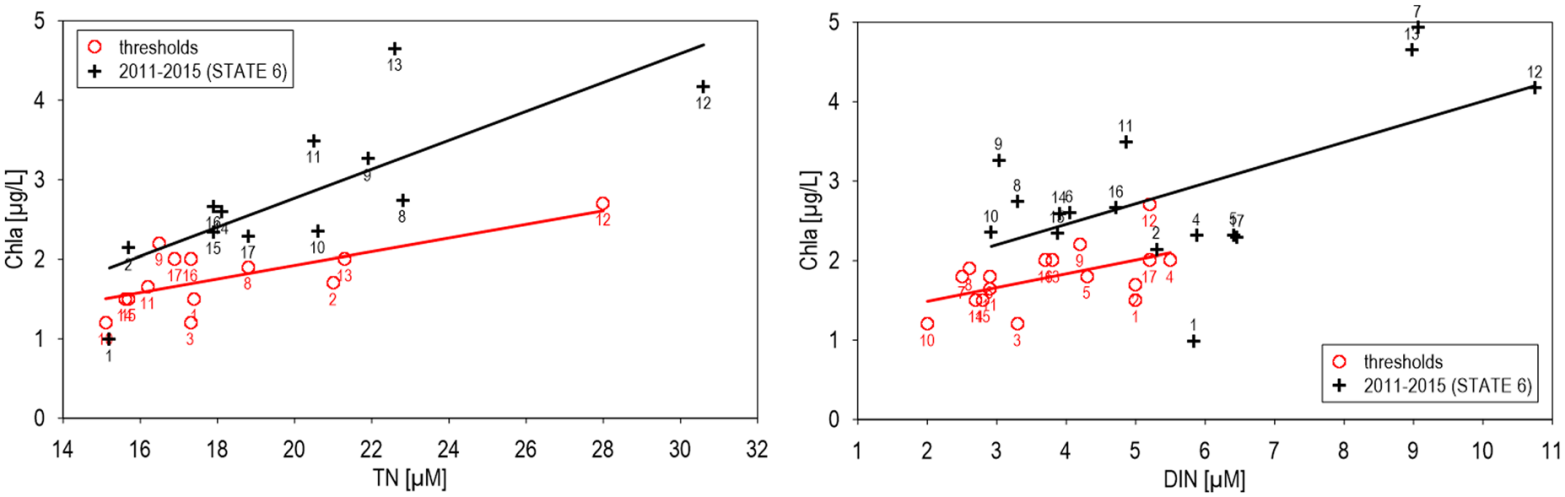
Correlations between chlorophyll-a and nutrients in the Baltic Sea (Basin means), thresholds, and recent data (2011–2015, STATE 6 Indicator reports, HELCOM, 2017). TN-Chla: Y = 0.085 × X + 0.215, n = 13, R^2^ = 0.509, α > 5%, Y = 0.258 × X + 1.421, n = 12, R^2^ = 0.592, α < 5%, DIN-Chla: Y = 0.173 × X + 1.142, n = 17, R^2^ = 0.285, α > 5%, Y = 0.1822 × X + 0.878, n = 16, R^2^ = 0.347, α > 5%

Correlations between local chlorophyll-a and nitrogen concentrations (Figs. 5 and 6) indicated significant relations of recent data and thresholds in the North Sea for DIN and similar tendencies in the Baltic Sea for basin means of DIN and TN. In the North Sea, chlorophyll-a was correlated significantly with DIN during winter, including thresholds, applied during different seasons (Fig. 5). In relation to DIN concentrations, chlorophyll-a thresholds were defined higher than in recent data combinations. The most strong deviations of thresholds from means were observed for inshore thresholds applied in the Netherlands, surpassing means by 490% and recent relations by 640% significantly. In coastal waters of the Netherlands and Belgium, chlorophyll-a thresholds surpassed mean thresholds and recent means significantly.

In contrast to relations between recent and threshold chlorophyll-a/DIN correlations in the North Sea, chlorophyll-a/recent TN and DIN relations (Fig. 5) were higher than threshold correlations in the Baltic Sea (Fig. 6). Relations between chlorophyll-a and TN concentrations were similar to those of DIN in the Baltic Sea, at a higher level of TN concentrations. Deviations of chlorophyll-nutrient relations within the different basins from mean relations remained moderate due to application of means, smoothing variability of local thresholds as a first step of assessment harmonisation. Especially for area 12 (Bay of Riga), chlorophyll thresholds were mostly higher than mean threshold fits and often similar to recent data combinations (Table 1).

## Discussion

Two widely used indicators for elevated nutrient loads in surface waters, nitrogen concentrations and phytoplankton biomass, measured as chlorophyll-a concentrations, were used to check the consistency of applied thresholds in regional eutrophication assessments in the North Sea and Baltic Sea regions (HELCOM, 2017; OSPAR, 2017). Nitrogen as the most limiting nutrient in coastal waters (Howarth & Marino, 2006; Loebel, 2009; Smith, 2006) and phytoplankton as the first parameter responding to elevated nutrients have been selected as examples for mixing diagrams and parameter correlations. Correlations of recently applied thresholds revealed consistent mean relations basically, within mixing diagrams and correlations between chlorophyll-a and nutrients, corresponding to similar tendencies for recent conditions (Figs. 1 and 2). These significant correlations allowed direct comparisons between slopes for recent data and thresholds.

Mixing processes in coastal waters mainly control nutrient gradients between river discharges and offshore waters, as indicated by significant linear mixing diagrams for DIN (Fig. 1). In the south-eastern North Sea, the mixing of rivers with estuaries and coastal waters was modulated by lateral transport by the continental coastal current, collecting and combining nutrient discharges along the southern North Sea coasts (Blauw et al., 2006; Otto et al., 1990). The Baltic Sea, representing an extended estuary, connected with the North Sea by mixing events and annual water exchanges (Feistel & Nausch, 2008), was characterised by dilution of river discharges as well, indicated by consistent tendencies of mixing diagrams with decreasing nutrient concentrations at increasing salinities (Fig. 3).

Nutrient thresholds of coastal areas are key values for regional assessments, because eutrophication in coastal waters is mainly caused by elevated nutrient discharges of rivers, which can be estimated as the freshwater end-members of mixing diagrams. River nutrient concentrations are dependent on discharge rates which are related to catchment area sizes and correlated with nutrient loads (Smith, 2005), offering similar conditions for definitions of consistent river thresholds. Mean North Sea DIN thresholds, approaching freshwater in the mixing diagram (Fig. 1), remained with 80.6 µM DIN a factor of 3 below recent means and a factor of 18 above natural background concentrations of 4.4 µM DIN (Topcu et al., 2011).

Differences between local thresholds, means, and recent data in mixing diagrams (Figs. 1 and 3) reflected clearly deviations even for means of Baltic Sea basins (HELCOM, 2017; OSPAR, 2017). Thresholds in open coastal waters, connected by a steady coastal current (Otto et al., 1990), should be similar and not surpassing mean thresholds by a factor of 2 in the Netherlands and Belgium (Fig. 1), considering the required harmonisation of assessments. This concerns as well the significant differences of applied DIN thresholds across assessment borders of coastal waters between Germany and the Netherlands and offshore between Germany with 7.45 µM DIN and the Netherlands and UK applying both 15 µM (Fig. 2). By the applied elevated offshore thresholds for DIN by the Netherlands and UK, surpassing mean thresholds by 21.6% (Fig. 1), recent mean offshore concentrations were surpassed by 74.4% within large areas of the North Sea, ignoring the recently (2006–2014) arrived steady state offshore of 8.6 µM DIN reflecting unlimited dilution and reduced atmospheric nitrogen deposition (Bartnicki et al., 2017).

For the Baltic Sea, the relationships between nutrient concentrations and salinities were most evident between the Bothnian Bay (area 17) and the Kattegat (1), reflecting regional effects of elevated river discharges in the Bothnian Bay similar to the Gulf of Riga (12) and the Gulf of Finland (13) (Fig. 3). Also, thresholds in the Baltic Sea can potentially be improved during longer time scales by relations to salinity (Fig. 4). Combined TN thresholds of basin means reached at freshwater conditions already 19.5 µM which is only 11.4% above natural background concentrations of 17.6 µM TN (Topcu et al., 2011) and recent basin means of 22.9 µM TN surpassed the background only by 31%. Most significant deviations of basin means from mean TN thresholds in the Gulf of Riga were related probably to recent elevated regional concentrations (Fig. 3), and local differences to the adjacent East Gotland Basin could be reduced by salinity-related correlations (Fig. 4 right). Thresholds of inshore and near coastal WFD areas, controlled by variable local mixing processes (tides, river fluctuations) reflecting mainly locally restricted effects, were not included within this approach, aiming to improve thresholds basically, but principally, by similar procedures, those thresholds could be improved as well.

By constant additions (e.g. 50%, OSPAR, 2008) to natural nutrient concentrations in rivers (Hirt et al., 2014; Topcu et al., 2011), validated by historical sediment analyses in the German Bight (Serna et al., 2010) or in a Baltic Sea fjord (Clarke, 2006; Clarke et al., 2003), applied fresh water thresholds could be harmonised (Claussen et al., 2009), avoiding diverging definition methods. Upper limits of freshwater thresholds are given by recent concentrations, correlated as area related nutrient discharges with the population density in catchment areas (Caraco, 1995; Howarth et al., 1996; Peierls et al., 1991) reflecting the degree of eutrophication modulated by effects of reduction measures. Between these boarders, deviations of thresholds from recent concentrations are a first approach for consistency checks and should be supplemented by relation to background data of mixing end-members represented by natural river concentrations and offshore concentrations with unlimited dilutions.

The often proposed constant 50% addition to background data (Almroth & Skogen, 2010; Claussen et al., 2009; HELCOM, 2017; OSPAR, 2008) cannot be applied to nitrogen concentrations offshore in central North Sea waters, because they were balanced by mixing of diluted coastal waters, the Atlantic inflow, atmospheric deposition, and denitrification (Topcu & Brockmann, 2015). For these reasons, offshore applied thresholds should not surpass recent (2006–2014) means, considering the fact, that these values characterise nearly natural conditions balanced by unlimited dilution, where nutrient concentrations can only be reduced by further reductions of atmospheric depositions (Bartnicki et al., 2017) or long-lasting reduction of elevated river discharges transferred by slow long-distance mixing. Therefore, national offshore thresholds in the North Sea above recent means of DIN (Fig. 1) are striking because offshore values are affected by transboundary transports as well (Otto et al., 1990) and cannot be managed by modified river discharges directly. For these reasons, offshore thresholds of DIN, applied by the Netherlands and UK during the recent assessment surpassing the mean mixing line and mean recent (2006–2014) offshore concentrations by 74% (Fig. 1), indicated significant inconsistent threshold definitions. For the open Baltic Sea, 20% addition to background data are proposed, based on main salinity gradients connected with the North Sea.

Effects of ongoing elevated anthropogenic atmospheric nitrogen depositions on recent offshore DIN-thresholds could be considered by reducing these values below present concentrations of mixing marine end-members, related to trends of decreasing atmospheric depositions (Bartnicki & Benedictow, 2017; OSPAR, 2009; Semena et al., 2015) or historical estimations (Laane, 1992; Prospero et al., 1996; Rendell et al., 1993; Ruoho-Airolaet al., 2012). Data for elevated atmospheric phosphorus deposition had been discussed as well but trends have not been quantified (Mahowald, 2008; Rolff et al., 2008). Surface water in the Sound, Kattegat/Skagerrak, and the Norwegian coastal waters, connected by the coastal current (Otto et al., 1990), was nearly DIN-depleted (Fig. 1), caused by the seasonally continuous primary production in the permanently stratified outflow of the Baltic Sea (Feistel et.al. 2008). This fact was reflected principally by low DIN thresholds. However, the applied thresholds were still about 100% above recent regional means, reflecting the need for consistency checks.

The areal distribution of thresholds, applied by OSPAR and HELCOM, highlight the artificial patchworks of independent local/regional threshold definitions (left Figs. 2 and 4), which are in contrast with natural gradients (right Figs. 2 and 4). However, based on correlations with salinity, harmonisations could be achieved according to recent salinity gradients. The patchwork of applied TN-threshold means for Baltic Sea basins requires further harmonisation of assessments by adaptation to salinity gradients (Fig. 4). Because TN concentrations are correlated with other eutrophication parameters, averaged basin-level TN concentrations, elevated by about 2 µM, may affect the complete regional assessment.

Based on the established relationships between nutrient concentrations and chlorophyll-a concentrations (Figs. 5 and 6), nutrient mixing diagrams can be a useful tool to check the applicability of defined nutrient thresholds (Greenwood et al., 2019; Topcu et al., 2009). Since nutrient gradients during growing season were similar to winter gradients, chlorophyll-a concentrations followed decreasing offshore gradients of nutrients, and correlations between chlorophyll during growing season and winter nutrients were both significant in the North Sea, allowing a direct application of seasonally defined thresholds. These correlations reflected mainly a regional all-year contamination by nutrients and ongoing winter production in shallow areas (Brockmann & Wegner, 1995; Zingone, 2010), increased by climate change. Eutrophication effects in coastal waters will be forced generally by climate changes due to extending growing seasons and increasing stratification, forcing primary production and succeeding oxygen depletion by degradation of organic matter (Doney, 2010; Rabalais et al., 2009; Topcu & Brockmann, 2015). Despite the non-significant correlations in the Baltic Sea, due to small salinity gradients and applied basin means (HELCOM, 2017), a similar relation between thresholds and recent data combinations was observed as in the North Sea, allowing direct comparisons of thresholds and assessed data.

Compared to the Baltic Sea, thresholds of chlorophyll-a in the North Sea were above recent conditions, indicating weak phytoplankton assessments. Locally elevated chlorophyll thresholds in relation to means and recent conditions were applied in the Netherlands, Belgium, and UK in inshore and coastal waters (Fig. 5) and required corrections because the significant correlations reflected mainly natural conditions (Nielsen et al., 2002; Smith, 2006; Tett et al., 2003). An especially high chlorophyll-a threshold, applied by the Netherlands in local inshore waters, deviated by a factor of 6.4 from regional mean threshold relations, was probably related to locally elevated values (Brockmann et al., 2018) and should be explained. Deviations of 125% from background, applied for WFD-chlorophyll-a thresholds in coastal waters (GIG, 2007), were based on local adaptations as well, neglecting salinity gradients and any natural connections and causing systematic contradictions to correlated assessed nutrients. Since these definitions are neither reproducible nor consistent, they should be harmonised at least by considering mixing gradients. Baltic Sea thresholds for chlorophyll indicated a more realistic approach in relation to nutrients, but high chlorophyll thresholds as in the Gulf of Riga (area 12) indicated adaptations to recent conditions, and deviations from mean thresholds in Eastern Gotland Basin (9) should be checked for consistency as well (Table 1).

Thresholds were also applied for definition of targets for river discharges, such as for the different Baltic Sea basins within the Baltic Sea Action Plan (HELCOM, 2007). Integrated HELCOM basin means, aimed to estimate allowable nutrient loads to the basins (HELCOM, 2007), did not indicate local deviations of thresholds from mean conditions. Improving consistency of thresholds by relation to modelled data (Almroth & Skogen, 2010; Schernewski, 2015) is less reproducible than relations to recent or natural background values (Topcu et al., 2006). Modelled data are more variable due to changes by repeated model improvements, forced by progressing developments, and are less reproducible due to often missing documentation of interim results, limiting comparisons of connected assessments. In the Baltic Sea, applied thresholds were hidden by the HELCOM HEAT (HELCOM eutrophication assessment tool) assessments based on a change-point analysis (< / > 1) by forming ratios between thresholds and recent means, reported as one value in spite of assessed value and applied threshold (Andersen, 2011; HELCOM, 2009). These ratios can neither be translated directly for comparisons of thresholds nor to local loads and their changes; however, backtracking the HEAT results to individual data points is possible (HELCOM, 2018a, 2018b, 2018c).

The presented parameters and correlations were taken as examples, because nutrients and chlorophyll-a, as indicators for phytoplankton biomass, were frequently monitored, and the relations of chlorophyll-a to nutrients are very indicative for eutrophication processes and the consistency of applied thresholds (Figs. 5 and 6). Consistency of thresholds for other eutrophication parameters can be achieved by similar correlations, based on causal relations between different nutrients, TN concentrations and Secchi depths (Fleming-Lehtinen & Laamanen, 2012), or for zoobenthos biomass, correlated with chlorophyll-a concentrations (Beukema et al., 2002). Mixing diagrams for other nutrients or parameters could be applied as well, improving the consistency of eutrophication thresholds.

## Recommendations and conclusions

Since applied thresholds showed similar correlations as recent data for DIN and TN, a suggested first step in defining consistent regional thresholds is the adaption to means from mixing diagrams and correlations between different causal linked parameters, such as between nutrients (TN and DIN) and chlorophyll-a, between TN and Secchi depths, or between chlorophyll-a and macro-zoobenthos.

- As the second step, thresholds should be related to natural background values (+ 50% for rivers, approaching 0% offshore in the North Sea, and 20% in the central Baltic Sea (related to salinity in the Baltic Proper).
- As the third step, deviations from background data should be reduced < 50% for rivers, considering climate change effects and oxygen depletion in coastal waters.
- Based on deviations from means and recent condition, it is indicated that a couple of thresholds (Table 1) should be checked again because of their contradictions to natural processes. Especially extreme or repeated deviations of different parameters for the same area should be corrected.
- Additionally, atmospheric depositions and extending primary production during winter due to climate change should be considered, by added N-equivalents to nitrogen values.
- Keeping the assessment procedure as transparent as possible and allowing detection of local hot spots or reduction effects, recent deviations from thresholds should be reported as % e.g. by mapping.

## Data Availability

Data are available at the cited sources; additional data can be requested from the corresponding author.
